# Implementing Common Metrics across the NIH Clinical and Translational Science Awards (CTSA) consortium

**DOI:** 10.1017/cts.2019.425

**Published:** 2019-11-26

**Authors:** Denise H. Daudelin, Laura E. Peterson, Lisa C. Welch, Redonna Chandler, Mridu Pandey, Farzad Noubary, Philip L. Lee, Harry P. Selker

**Affiliations:** 1 Tufts Clinical and Translational Science Institute, Tufts University, Boston, MA, USA; 2 Institute for Clinical Research and Health Policy Studies, Tufts Medical Center, Boston, MA, USA; 3 The National Institute on Drug Abuse, National Institutes of Health, Bethesda, MD, USA; 4 Clear Impact, LLC, Rockville, MD, USA

**Keywords:** Performance improvement, Common Metrics, translational science, Results-Based Accountability, CTSA

## Abstract

The Clinical and Translational Science Award (CTSA) Consortium and the National Center for Advancing Translational Science (NCATS) undertook a Common Metrics Initiative to improve research processes across the national CTSA Consortium. This was implemented by Tufts Clinical and Translational Science Institute at the 64 CTSA academic medical centers. Three metrics were collaboratively developed by NCATS staff, CTSA Consortium teams, and outside consultants for Institutional Review Board Review Duration, Careers in Clinical and Translational Research, and Pilot Award Publications and Subsequent Funding. The implementation program included training on the metric operational guidelines, data collection, data reporting system, and performance improvement framework. The implementation team provided small-group coaching and technical assistance. Collaborative learning sessions, driver diagrams, and change packages were used to disseminate best and promising practices. After 14 weeks, 84% of hubs had produced a value for one metric and about half had produced an initial improvement plan. Overall, hubs reported that the implementation activities facilitated their Common Metrics performance improvement process. Experiences implementing the first three metrics can inform future directions of the Common Metrics Initiative and other research groups implementing standardized metrics and performance improvement processes, potentially including other National Institutes of Health institutes and centers.

## Introduction

The Clinical and Translational Science Award (CTSA) Program was established by the National Institutes of Health (NIH) in 2006 to accelerate and improve clinical and translational research and is currently part of the NIH National Center for Advancing Translational Sciences (NCATS). Nationally, CTSA Consortium “hubs,”^1^ at academic health care institutions, are funded to provide core resources, essential mentoring and training, and opportunities to develop innovative approaches and technologies designed to re-engineer the nation’s clinical and translational research enterprise.

In 2013, a congressionally mandated Institute of Medicine (IOM) (now the National Academy of Medicine) report on the CTSA Consortium envisioned it as an integral part of a learning health care system “*founded on the concept of continuous improvement and the imperative to translate ‘what we know’ into ‘what we do’*.”^1^ To move toward realizing this vision, the IOM Committee recommended that NCATS undertake an evaluation process to determine the effectiveness and impact of individual CTSA Consortium hubs and the program as a whole.

The report suggested “common metrics” to enhance transparency and accountability related to the activities undertaken by the CTSA Consortium. In response to this recommendation, NCATS embarked on a collaborative process with the CTSA Consortium grantees to develop the Common Metrics Initiative. Subsequently, the NIH research strategic plan for Fiscal Years 2016–2020^2^ included a primary objective to excel as a federal science agency by managing for results. Seen as an important piece of this effort, the Common Metrics Initiative was made a high priority for NCATS and the CTSA Program.

This article describes the design and implementation of the first three metrics for the CTSA Consortium Common Metrics Initiative, which entailed the use of shared metrics and an improvement framework across the CTSA Consortium, to promote collaborative performance improvement.

## Methods

### CTSA Consortium

Starting in 2015, the Common Metrics Initiative was adopted across the CTSA Consortium and at each hub. Within the mission of promoting clinical and translational research, hub characteristics vary, including size, geographic location, patient populations, students, programmatic and translational science focus, number and complexity of stakeholder collaborations, and length of time they have received NIH funding.^3^

### Metric Development

The development of specific metrics was a collaborative process which included NCATS staff, CTSA Consortium leaders and their teams, CTSA Consortium Evaluators (representing those already doing evaluation at hubs), and outside consultants. In 2015, working groups comprised of CTSA Consortium leaders and staff, and NCATS staff met in a series of facilitated webinars. Four groups were formed to reflect key services provided by hubs, and the activities of these workgroups resulted in the selection of three initial metrics for implementation: (1) Median Institutional Review Board (IRB) Review Duration; (2) Careers in Clinical and Translational Research; and (3) Pilot Funding Publications and Subsequent Funding. Each metric, as well as its specifications and key definitions (“Operational Guidelines”), was developed by a group of topic-specific experts, evaluators, CTSA hub principal investigators (PIs), and NCATS staff. This process included reviewing relevant literature, discussions with other academics and across NIH, and group meetings. After development and a pilot test by four volunteer hubs, a revised Operational Guideline template (Supplementary Table S1) was developed, and metric implementation by the remaining hubs used postpilot versions of the metrics and Operational Guidelines.

### Performance Improvement Methodology

The Common Metrics Initiative adopted and is currently guided by the Results-Based Accountability (RBA) improvement framework,^4^ which is used by programs, organizations, and multiorganizational efforts to measure and improve performance and enhance impact. In the CTSA Consortium, the metrics provide data related to performance and inform a “Turn the Curve” (TTC) performance improvement plan. The Common Metrics are intended to serve as a starting point for a larger effort to understand the data within the context of the local hub environment and to develop strategies to improve performance. Thereby, the Common Metrics results can assist in assessing whether the CTSA Consortium’s resources are being optimally used to develop and deliver services and resources – at individual hubs and Consortium-wide.

### Initiative Organizational Structure and Oversight

Between June 2016 and December 2017, an implementation team from the Tufts Clinical and Translational Science Institute (CTSI) Research Process Improvement Program led the roll-out of the first three Common Metrics. Bi-weekly meetings were held with NCATS representatives, the Tufts Implementation Team, an RBA consultant, and the Tufts CTSI PI to address implementation issues and track progress. A Common Metrics Executive Committee comprised of CTSA PIs, CTSA hub program evaluation and administrative leadership staff, NCATS CTSA Program staff, an RBA consultant, and Tufts Implementation Team representatives, met monthly to build buy-in and stakeholder engagement across the CTSA Consortium.

### Implementation Activities

The Implementation Team supported 64 CTSA hubs in implementing metric data collection, the use of the RBA improvement framework, and a supporting web-based data reporting and communication system (“Scorecard”). The implementation was not considered human subjects research by the Tufts Health Sciences IRB.

### Hub Common Metrics Teams

Each CTSA hub was asked to form a “core” Common Metrics team covering five roles:

- *Project Champion –* ensured everyone at the hub was “on-board” and committed to the project’s success.
- *Project Leader* – responsible for overall project planning and execution.
- *RBA Framework Lead* – responsible for helping their team learn and implement the RBA framework.
- *Scorecard Software Lead* – responsible for helping others at the hub learn the Scorecard software.
- *Metrics Topic Lead* – a subject matter expert responsible for overseeing metric data collection. Hubs could elect to have a different metric expert for each Common Metric.

Hubs were also instructed to identify additional subject matter experts to assist with data collection and performance improvement for specific metrics (e.g., a member of the institution’s IRB for the IRB Review Duration metric).

### Training

Hub teams participated in training and subsequent coaching in one of three implementation groups based on hubs’ preferences. Initially, each group received online training on the metrics, software, and performance improvement concepts. The webinar training sessions included:

- *“Onboarding” session*: A review of the Common Metrics project activities and timeline and guidance on finalizing the hub’s team.
- *Common Metrics Training:* A review of the Operational Guideline for each metric.
- *PI Training:* A review of the RBA framework and Scorecard software for CTSA hub PIs.
- *RBA and Scorecard Training:* Three sessions for hub team members using a mix of prerecorded lectures, training videos, and live interactive webinars to build knowledge and skills in the RBA framework and in using the Scorecard software.
- *Kickoff session:* This session provided the full hub team project timeline updates, examples of completed Scorecards, and guidance on next steps.

Based on participant feedback, the training was modified after the first and second implementation groups. Interactivity was increased, and CTSA-specific examples were added.

### Coaching

After training, each hub selected one of the three Common Metrics for the first phase of data collection and TTC performance improvement activities. Each Implementation Group was divided into smaller coaching groups of four to six hubs. During seven every-other-week small group coaching sessions, a quality improvement consultant from the Tufts Implementation Team facilitated a 1-hour webinar during which hubs discussed their progress, challenges and barriers in collecting metric data according to the Operational Guidelines, and developing TTC plans for performance improvement. Hubs could ask questions about defining terms, data sources, and how to apply the Operational Guidelines in specific situations.

It was the expectation that for one Common Metric, hubs would complete data collection, metric computation and data entry to Scorecard, and an initial TTC plan by the conclusion of the their respective Implementation Group’s coaching period. At that point, hub teams were instructed to move on to data collection and TTC planning for the second and third Common Metrics. The Implementation Team remained available for technical support upon request.

### Disseminating Best and Promising Practices

As hubs began performance improvement, the Implementation Team convened a series of collaborative Learning Sessions to showcase and share emerging best and promising improvement strategies. All hub team members were encouraged to attend these optional 1-hour webinars of didactic presentations and group interaction.

Initial versions of a driver diagram and change package were also developed for each metric. A driver diagram is an evidence-based resource used in improvement efforts,^5^ designed around an aim or a team’s objectives. Drivers, factors that, if present, can help achieve the aim, along with potential strategies for improving performance, were identified through a literature review and initial TTC plans. The change packages included example strategies from hubs for each driver, including a brief rationale.

### Technical Assistance

The Implementation Team provided ongoing technical assistance to hubs to help them: (a) interpret and apply the Operational Guidelines; (b) identify and overcome barriers to data collection and performance improvement; (c) complete TTC plans; and (d) locate and use resources developed by the Implementation Team. A website provided a range of initiative-related resources, including answers to frequently asked questions, training materials for RBA, Scorecard, and the individual metrics, and slides and video recordings of the Learning Sessions.

Training materials included metric calculation worksheets and example improvement plans. Despite guidance in the Operational Guidelines, hubs had numerous questions and misunderstandings about how to calculate metric score values. They also questioned the level of detail that should be included in an initial TTC plan. Therefore, the Implementation Team developed: (1) worksheets for both the Pilot Publications and the Careers Common Metrics with examples and tools to help calculate each metric score and (2) an example TTC plan for the Careers Metric.

Technical assistance also addressed data quality. The Implementation Team performed an iterative metric value checking process and provided feedback to hubs when metric scores were out of anticipated ranges or were obviously inconsistent with the Operational Guidelines.

Finally, a set of annotated slides, based on the initial training, was provided to allow core team members to train additional staff at their hub.

### Data and Analyses

Hub participation in training and coaching activities was tracked by Implementation Team staff and the webinar log-in software. Attendance was defined as at least one person from the hub logging into the webinar.

Hub progress was assessed by whether a hub produced a metric value (yes/no) and the extent to which its TTC plan met criteria for applying the RBA framework. Prior to each coaching webinar, the quality improvement consultants reviewed metric values and TTC plans entered by hub teams into Scorecard. Implementation Team staff took detailed notes during each webinar. After each webinar, progress on TTC planning for each hub was assessed by the quality improvement consultants based on 13 criteria, using a structured tool (Supplementary Table S2). These assessments also helped identify content for upcoming calls and needs for individual hub support.

Hub experiences and challenges were summarized based on discussions with hubs during coaching activities and requests for assistance. In addition, as part of an independent formal evaluation study of the Common Metrics Implementation (reported separately), all hubs received an online, self-report survey designed in part to assess the implementation activities. Administered in January–February 2018, the survey addressed, in part, the amount and time allocated to implementation program components, measured on a five-point Likert-type scale (“much less than was needed” to “much more than was needed”). An invitation email was sent to one PI per hub, who was instructed to assign one person to complete the survey with input from others at the hub. The survey was completed by 59 of the 60 hubs (98%) receiving it.

Quantitative data were summarized using frequencies and proportions for categorical variables and using means and standard deviations for continuous and count variables. Means for Implementation Groups were compared using an analysis of variance model and *p*-values from overall *F*-tests.

## Results

### Implementation Groups and Choice of Common Metric

All hubs were assigned to their first or second choice of Implementation Group. The number of hubs per group and their choice of metric on which to focus during the coaching period are depicted in Table 1. Hubs reported various rationales for their choice of Implementation Group and metric. Some selected Implementation Group 1 to have adequate time to work on metrics they perceived would require more effort. Many selected a metric for which they had preexisting data, either from previous improvement efforts or because they assessed a similar metric on an ongoing basis (e.g., IRB turnaround time). Others reported choosing a metric they assumed would be harder for them to collect data for, or a metric topic for which they had fewer identified partners, believing that determining a metric value and/or developing a TTC plan would take more time.

**Table 1.** Number of hubs and self-selected coaching metric by Implementation Group

Implementation group	*n*	First Common Metric		
		Median IRB Review Duration *n* (%)	Careers in CTR *n* (%)	Pilot Funding Publications *n* (%)
Pilots	4	0 (0)	2 (50)	2 (50)
Implementation Group 1	20	8 (40)	5 (25)	7 (35)
Implementation Group 2	18	7 (39)	5 (28)	6 (33)
Implementation Group 3	22	9 (41)	3 (13)	10 (46)
Total	64	24 (38)	15 (23)	25 (39)

IRB, Institutional Review Board; CTR, Clinical and Translational Research.

### Hub Team Composition and Participation in Training and Coaching

Hubs configured their teams based on their current staffing. At some hubs, the same person assumed more than one role (such as project leader or RBA expert) or two people shared the same role (e.g., project coleaders). The resulting teams varied in size (range: 2–11). Some teams had broad membership, whereas in a few, one person, the “Common Metrics person,” conducted virtually all activities. Additionally, teams often lacked participation of additional subject matter expert members with specialized knowledge of metric topics or with influence over relevant operational areas.

There was wide participation in the training and coaching and, on average, at least one person from each hub attended most sessions (Table 2). Compared to other groups, team members from Implementation Group 1 attended slightly more training sessions, and team members from Implementation Group 2 attended slightly fewer coaching sessions.

**Table 2.** Hub participation in training and coaching, overall and by Implementation Group (*n* = 59*)

Characteristic	All hubs	Implementation Group			*p*-value
		1 (*n* = 20)	2 (*n* = 17)	3 (*n* = 22)	
Program attendance** (mean, SD)					
Training sessions (*n* = 7)	6.3 (1.10)	6.9 (0.37)	6.1 (0.75)	6.0 (1.54)	**0.02**
Coaching sessions (*n* = 6***)	5.6 (0.75)	5.7 (0.67)	5.2 (1.01)	5.9 (0.35)	**0.01**

* Excluding four pilot hubs and one hub not responding to the survey.

** Attendance at a training or coaching session is defined as at least one person from the hub attended.

*** Based on six coaching sessions, Implementation Groups 1 and 2 were offered seven sessions; Implementation Group 3 was offered six sessions.

### Evaluation of Training

**Fig. 1.**
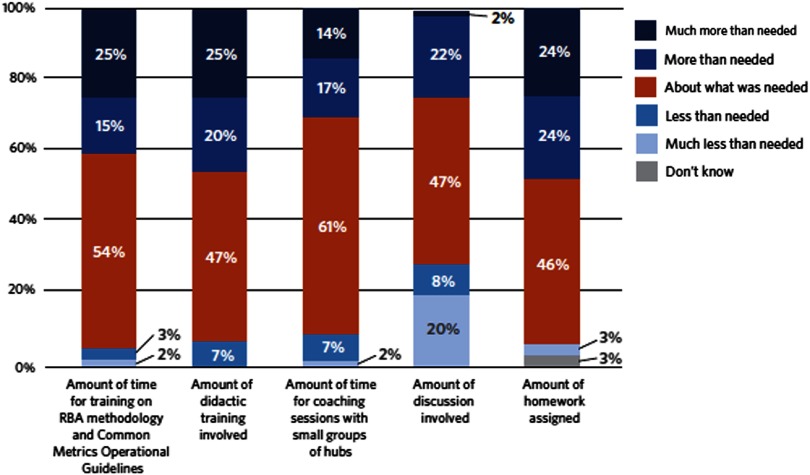
Hub evaluation of implementation training and coaching. RBA = Results-Based Accountability.

Hub responses to questions about the amount of time allocated for training on RBA and the metric Operational Guidelines and ratings of the didactic training overall are summarized in Fig. 1. There were no statistically significant differences by the Implementation Group despite changes over time in response to hub team feedback. Hubs reported that the didactic webinars were professionally presented but that sessions included redundant information. Hubs also reported they would have preferred that the training had taken less time and included more relevant examples.

### Evaluation of Coaching, Discussion, and Homework

There were no statistically significant differences in the evaluation of coaching, discussion, and homework by Implementation Group. Hub responses to questions about the amount of time allocated for coaching sessions and the ratings of the amount of discussion and homework are summarized in Fig. 1. Hubs generally found the coaching sessions supported implementing the Common Metrics as they facilitated sharing experiences with and successful strategies for implementation across hubs. Hubs pointed to the opportunity to share experiences with each other as key. Some hubs indicated a desire to continue with coaching sessions. Others indicated that the intensity and frequency of sessions had been crucial initially, but that the same intensity was not necessary over time.

### Hub Progress

Hubs progressed at variable rates in all components of the Common Metrics Implementation. Although many hubs had been collecting data prior to metric implementation, for some hubs and some metrics, existing data sources did not align with Operational Guidelines or access to the data was limited. Developing new data sources, revising existing sources, or gaining access to data led to delays in collecting and reporting metric values. By the conclusion of their respective coaching periods, 54 of 64 hubs (84%) had produced a value for the metric they focused on during coaching. However, some of the values were inconsistent with the Operational Guidelines.

**Fig. 2.**
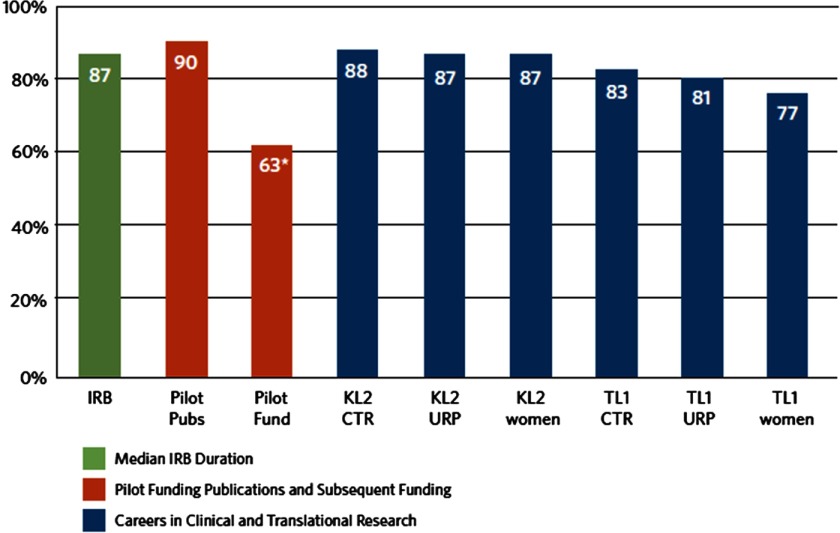
Percent of hubs that produced a metric value by the end of the coaching period. *Optional metric; IRB = Institutional Review Board; KL2 = Career Development Award Program; TL1 = Pre- and Postdoctoral Training Program; URP = under-represented persons.

Many hubs also experienced challenges in completing one or more elements of the RBA framework. Approximately half of hubs met the Implementation Team’s assessment criteria for four RBA components: Story Behind the Curve (56%), Engaging Partners (40%), What Works (53%), and Strategies (50%) (Fig. 2). Several criteria related to the development and implementation of the TTC plan (e.g., developing action plans) were unable to be fully assessed during the coaching period. However, the quality improvement consultants expected that continued progress would be made after the coaching period ended. Multiple factors affected the pace and quality of developing TTC plans during the coaching period. Facilitating factors included having conducted previous improvement efforts on the topic and having a perception that Common Metrics activities were synergistic with other hub priorities. Barriers included limitations of access to data to compute the metric or analyze the results; availability and engagement of project team members, partners, and subject matter experts; and perceived usefulness of the metric. Some hubs were reluctant to develop plans until metric values were available; others concentrated their plans on implementing or revising data sources. When hubs believed their current level of performance did not require improvement, their improvement plans primarily described activities they had previously undertaken relative to the metric topic.

## Discussion

To our knowledge, this is the first implementation of performance metrics and a process improvement framework across a national research consortium. In partnership with NCATS, the Tufts Implementation Team developed and delivered a program of activities intended to provide the CTSA Consortium and its hubs with tools to support full adoption of collaborative performance measurement and improvement. Three metrics were developed by NCATS staff and CTSA Consortium leaders and staff. The Tufts Team provided all hubs training, coaching, and technical assistance on metric data collection and performance improvement. Collaborative learning sessions, driver diagrams, and change packages were used to disseminate best and promising practices. The majority of hubs produced a value for a first metric and about half produced an initial performance improvement plan by the conclusion of their respective coaching period. Overall, hubs reported that the implementation activities facilitated their Common Metrics performance improvement process.

The results of implementing these first three metrics can inform decision-making on future directions of the Common Metrics Initiative. The lessons learned may also be of interest to other research groups or networks implementing standardized metrics and performance improvement processes, potentially including other NIH institutes and centers.

CTSA organizations and their personnel are very diverse. Accordingly, hubs were heterogeneous in their approach to, and engagement with, the Common Metrics Implementation. They varied in their perceptions of metric usefulness at the local and national level. Common Metrics did not always align with hub institutional priorities and were not consistently viewed as useful for local improvement. Getting input from and expanding pilot testing to include insights from broader groups of hub staff and stakeholders could help metrics have greater utility to hubs. Although this initial implementation was generally positively received, having metrics that are closer to hubs’ specific objectives will no doubt further increase support for, and engagement in, shared metrics and performance improvement activities.

Hubs also expressed a wide range of organizational and participant needs. Optimally, if resources allow, training and coaching should be customized, providing options to meet the varying needs of adult learners and various levels of prior performance improvement experience. Coaching is fundamental to implementing metrics and performance improvement. Coaching can: (1) promote accountability to meet implementation milestones; (2) help assess implementation progress; (3) identify needs for implementation assistance; and (4) connect participants with peers for better identification of barriers and facilitators. Therefore, small group coaching was useful when implementing each new metric. Additional optional coaching could be provided after initial metric implementation.

The initial training aimed to provide hub team members basic proficiency in RBA, which was new to the majority. Over time, as participants become more proficient in creating performance improvement plans, additional training on more advanced performance improvement concepts could be provided.

Metric implementation could also benefit from greater emphasis on including metric-specific team members and addressing factors that facilitate effective improvement teams. Including more partners and active subject matter experts beyond the core team would help teams gain a deeper understanding of the underlying causes of existing performance, address data issues, and identify, select, and implement effective metric-specific improvement strategies. Team functioning could be heightened by identifying one team member who “owns” the project and including a local champion on the team.^6^ Active involvement of the CTSA PI is essential. Some teams may also need mechanisms to promote accountability for meeting milestones.

To fully realize the potential of the Common Metrics Initiative, or similar initiatives, ongoing attention should be given to metric data quality and completeness. This is particularly true to ensure data comparability for cross-consortium comparisons or benchmarking. Hubs require time to realign existing information systems and other infrastructure or to build new data collection systems if needed. Given the detailed Operational Guidelines, and the heterogeneity of hub data sources and systems, concrete examples of how to calculate the metrics should be developed for each new metric.

Finally, to achieve optimal benefit from the resources necessary for metric development and implementation requires a parallel emphasis on applying the performance improvement framework. CTSA hubs need to ensure that they understand the underlying causes behind current performance, prioritize the multiple opportunities for improvement, and recognize performance barriers and facilitators. They must identify a range of potential strategies, and select those that are actionable, impactful, and consistent with their mission and goals. They must also be sensitive to resource requirements, costs, and benefits. Over time, an emphasis on action planning and on repeated measurement to determine program effectiveness at both the local hub and CTSA Consortium levels will support the Common Metrics Initiative in achieving its objectives.

## Supplementary material

For supplementary material accompanying this paper visit http://dx.doi.org/10.1017cts.2019.425. click here to view supplementary material
