# Supplementary material for: Implementing Common Metrics across the NIH Clinical and Translational Science Awards (CTSA) consortium
Source: J Clin Transl Sci. 2019 Nov 26;4(1):16–21. doi: 10.1017/cts.2019.425 (PMC7103469; doi:10.1017/cts.2019.425)
Supplement: Supplementary file 1 [file S2059866119004254sup.zip › S2059866119004254sup001.docx]

**Table S1. Template for Common Metric Operational Guidelines**

| **Template Element** | **Description** |
| --- | --- |
| 1. Operationalized Metric Title | *[Name of Metric as it should be implemented.]* |
| 1. Rationale | *[An explanation that states why it is important to collect data/information on this measure. This may include specific literature references, evidence based information, expert consensus, etc.]* |
| 1. Operational Specification | *[Description of metric being measured.]* |
| 1. Technical Description | *Key Definitions*  *Timeframe*  *Data Scope* |
| 1. Metric Type, Numerator and Denominator Statements or Continuous Variable Statement, Inclusion/Exclusion Criteria | *[Metric Type (e.g., count, rate, proportion, mean, or median]*  *Numerator & Denominator Statements or Continuous Variable Statement (based on Metric Type) with Inclusion & Exclusion Criteria*  *Note: If the measure is reported as a rate (proportion or ratio), the Numerator and Denominator*  *Statement are completed. If a performance measure does not have both a numerator and a denominator, then a Continuous Variable Statement is completed.*  *Denominator Statement*  *Represents the population evaluated by the performance measure.*   - ***Inclusion Criteria for Denominator:*** *Specific information describing the population(s) comprising the denominator, not contained in the denominator statement or not applicable* - ***Exclusion Criteria for Denominator:*** *Specific information describing the population(s) that should not be included in the denominator, or none*   *Numerator Statement*  *Represents the portion of the denominator population that satisfies the conditions of the performance measure to be an indicator event.*   - ***Inclusion Criteria for Numerator:*** *Specific information describing the population(s)*   *comprising the numerator, not contained in the numerator statement, or not applicable*   - ***Exclusion Criteria for Numerator:*** *Specific information describing the population(s) that*   *should not be included in the numerator, or none*  *Continuous Variable Statement*  *Describes an aggregate data measure in which the value of each measurement can fall anywhere along a continuous scale.*   - ***Inclusion Criteria for Continuous Variable:*** *Specific information describing the population(s) comprising the performance measure, not contained in the continuous variable statement or not applicable* - ***Exclusion Criteria for Continuous Variable:*** *Specific information describing the population(s) that should not be included in the performance measure or none* |
| 1. Data Sources & Methods of Data Collection | *[Data Sources, Method of Data Collection]* |
| 1. Frequency of data collection and Reporting | *[Frequency of data collection and reporting.]* |
| 1. Unit of Analysis | *[Unit of Analysis]* |
| 1. Notes/Comments | *[Additional notes/comments]* |
