# Supplementary material for: Implementing Common Metrics across the NIH Clinical and Translational Science Awards (CTSA) consortium
Source: J Clin Transl Sci. 2019 Nov 26;4(1):16–21. doi: 10.1017/cts.2019.425 (PMC7103469; doi:10.1017/cts.2019.425)
Supplement: Supplementary file 1 [file S2059866119004254sup.zip › S2059866119004254sup002.docx]

**Table S2: Hub Assessment Criteria**

| ***Criterion*** | ***1-Not Meeting*** | ***2-Approaching*** | | ***3-Meeting*** | **4-*Exceeding**** |
| --- | --- | --- | --- | --- | --- |
| 1. **Team formation, training, and stakeholder involvement** | | | | | |
| A core team of at least 2 individuals is formed at hub and members participate in training. Core team trains other hub staff. | No participation in training | A key role did not participate in training. Little or no training of hub staff | | All key roles participated in training. Training conducted for 1-2 hub staff based on hub needs. | Broad participation in training across core team and hub staff |
| Relevant stakeholders/ metric topic experts are identified and engaged in the Turn the Curve (TTC) process | No stakeholder, topic expert involvement beyond core team | Ad hoc involvement of 1-2 stakeholders/ metric topic experts | | Regular involvement of stakeholders/ metric topic experts | Broad involvement of >2 stakeholders/ metric topic experts |
| 1. **“How are we doing?”** | | | | | |
| Common Metric (CM) data are collected and entered to the online Scorecard system | No CM data have been entered | Estimated values for current CM data have been entered | | Actual values for current CM data have been entered | Historical values for CM data have been entered |
| The graph includes a baseline forecast (i.e., that assumes no new actions) | No baseline forecast | A baseline forecast is present but incorrect | | A baseline forecast is present |  |
| 1. **“What is the story behind the curve?”** | | | | |  |
| Identifies the factors/underlying causes that influence the value of the performance measures (includes factors that are positive and negative, internal and external, current and anticipated) | No or minimal number (*e.g*., 1-2) of factors identified. No exploration to identify root causes. | | Factors are identified but not in a range of categories. Partial exploration to identify root causes. | Factors are identified in a range of categories. Exploration to identify root causes. | Multiple factors are described in multiple categories.  Complete exploration to identify root causes. |
| Factors are prioritized according to strength of influence (“leverage”) | Factors are not yet identified | | Factors are identified but priorities are incomplete or unclear | Clearly prioritizes factors | Rigorous process for prioritizing factors |
| 1. **“Who are partners who might have a role to play in turning the curve?”** | | | | |  |
| Identifies partners who might have a role to play in turning the curve (e.g., people who could affect change) | No partners or roles are identified | | Relatively few partners and roles identified | Appropriate number and roles of partners identified | Extensive involvement of partners |
| 1. **“What works to turn the curve?”** | | | | |  |
| Identifies potential actions (including low-cost and/or no cost actions) related to high priority factors identified in the story behind the curve (SBtC) | No actions identified or a few actions that are not related to priorities in SBtC | | Actions present but unclear relationship to priorities in SBtC and/or do not include low-cost or no cost items | Actions and priorities in SBtC are clearly related. Actions include low-cost or no cost items. | Innovative actions identified |
| Identifies where additional information will be needed to sufficiently understand what works to turn the curve | No description of adequacy of available information | | Additional information needed but no plan to gather | All needed information available or plan to gather is described | Identifies and implements plan for additional data |
| 1. **“What do we propose to do to turn the curve?”** | | | | |  |
| Actions are selected related to high priority factors identified in the story behind the curve | No actions are present or they are not related to high-priority factors | | Actions are present but unclear how or whether the actions are related to high-priority factors | Actions are present that are clearly related to high-priority factors | Actions for lesser factors as well as high priority factors are identified |
| Action plans provide specifications necessary to be implemented (*e.g*., deliverables, person(s) responsible, necessary resources, and deadlines) | No action plans or action plans are vague | | Action plans are insufficiently detailed for implementation | Action plans are clear with amount of detail necessary for implement-ation | Action plans are clear, strong project management structure in place |
| **7. Selecting performance measures (PMs) for managing a component of a strategy to turn the curve of a Common Metric** | | | | | |
| PMs are clearly defined | No PMs defined | | Incomplete measure definition | Complete and clear measure definition | Data collection for PMs has begun |
| PMs have TTC plans | No turn-the-curve plan associated with PMs | | Partial TTC plan developed | Full TTC plan developed | Plan for ongoing management developed |

*The rating of “Exceeding criteria” was included to identify hubs whose TTC plan demonstrated best practices in applying the RBA framework.
